# Hsf1 Activation Inhibits Rapamycin Resistance and TOR Signaling in Yeast Revealed by Combined Proteomic and Genetic Analysis

**DOI:** 10.1371/journal.pone.0001598

**Published:** 2008-02-13

**Authors:** Sricharan Bandhakavi, Hongwei Xie, Brennon O'Callaghan, Hiroshi Sakurai, Do-Hyung Kim, Timothy J. Griffin

**Affiliations:** 1 Department of Biochemistry, Molecular Biology and Biophysics, University of Minnesota, Minneapolis, Minnesota, United States of America; 2 School of Health Sciences, Faculty of Medicine, Kanazawa University, Kanazawa, Ishikawa, Japan; Wellcome Trust Sanger Institute, United Kingdom

## Abstract

TOR kinases integrate environmental and nutritional signals to regulate cell growth in eukaryotic organisms. Here, we describe results from a study combining quantitative proteomics and comparative expression analysis in the budding yeast, *S. cerevisiae*, to gain insights into TOR function and regulation. We profiled protein abundance changes under conditions of TOR inhibition by rapamycin treatment, and compared this data to existing expression information for corresponding gene products measured under a variety of conditions in yeast. Among proteins showing abundance changes upon rapamycin treatment, almost 90% of them demonstrated homodirectional (i.e., in similar direction) transcriptomic changes under conditions of heat/oxidative stress. Because the known downstream responses regulated by Tor1/2 did not fully explain the extent of overlap between these two conditions, we tested for novel connections between the major regulators of heat/oxidative stress response and the TOR pathway. Specifically, we hypothesized that activation of regulator(s) of heat/oxidative stress responses phenocopied TOR inhibition and sought to identify these putative TOR inhibitor(s). Among the stress regulators tested, we found that cells (*hsf1-R206S, F256S* and *ssa1-3 ssa2-2)* constitutively activated for heat shock transcription factor 1, Hsf1, inhibited rapamycin resistance. Further analysis of the *hsf1-R206S, F256S* allele revealed that these cells also displayed multiple phenotypes consistent with reduced TOR signaling. Among the multiple Hsf1 targets elevated in *hsf1-R206S, F256S* cells, deletion of *PIR3* and *YRO2* suppressed the TOR-regulated phenotypes. In contrast to our observations in cells activated for Hsf1, constitutive activation of other regulators of heat/oxidative stress responses, such as Msn2/4 and Hyr1, did not inhibit TOR signaling. Thus, we propose that activated Hsf1 inhibits rapamycin resistance and TOR signaling via elevated expression of specific target genes in *S. cerevisiae*. Additionally, these results highlight the value of comparative expression analyses between large-scale proteomic and transcriptomic datasets to reveal new regulatory connections.

## Introduction

Understanding how organisms respond to multiple environmental cues to adjust cellular growth and organismal development has been a long standing aim of biology. Recent work has revealed that the TOR (Target Of Rapamycin) kinases play an evolutionarily conserved central role in this integration (for recent reviews, see [1]–[3]). The TOR proteins are members of the phosphatidylinositol kinase (PIK) family of kinases. Unicellular fungi such as *S. cerevisiae* harbor two homologous TOR genes, Tor1 and Tor2, whereas higher organisms contain only one TOR gene. Much of our understanding of the TOR kinases has come from use of the bacterially derived drug, rapamycin, which specifically inhibits one of the two TOR kinase complexes, TORC1. Owing to the role of TORC1 complex in regulation of cell growth and the specificity of rapamycin, the drug (or its derivatives) is currently used in antirestenosis, antifungal, and immunosuppresant treatments in humans. TOR kinases also exist in a distinct TORC2 complex which has been implicated in the spatial control of cellular growth [4], [5].

Microarray analyses in yeast and human cells have demonstrated dramatic effects of rapamycin on gene expression [6]–[10]. Even though it is generally accepted that correlation between mRNA and protein levels is not always linear [11], [12], quantitative proteomic profiling of rapamycin treatment has not been done in any organism to date. In the case of a highly studied organism like the budding yeast, *S. cerevisiae*, microarray expression data for the entire genome under a variety of perturbations is available [13], and several global rapamycin fitness screens have been carried out [14]–[17]. Integration of these diverse datasets with the rapamycin-induced proteomic expression profile could potentially provide new insights into regulatory pathways that intersect with TOR signaling.

With the aim of gaining new insights into TOR function and regulation, we have performed quantitative proteomic profiling of yeast cells treated with rapamycin, in combination with comparative expression analysis of this data with existing microarray data in *S cerevisiae*. Of the proteins identified whose steady-state levels changed upon rapamycin treatment, we observed that a large majority of their corresponding mRNA transcripts also undergo a similar change under conditions of heat/oxidative stress. Because the known responses regulated by TOR did not fully explain this overlap, we hypothesized that activation of stress regulator(s) phenocopied TOR inhibition. Testing this hypothesis using genetic analysis, we found that constitutive activation of the conserved stress regulator Hsf1 confers rapamycin sensitivity and reduced TOR signaling via elevated expression of Hsf1 target genes. These findings identify Hsf1 as a putative inhibitor of TOR signaling and provide new insights into the relationship between stress signals and the inhibition of cell growth.

## Results

### Quantitative proteomics reveals changes in protein abundance induced by rapamycin treatment

**Figure 1:**
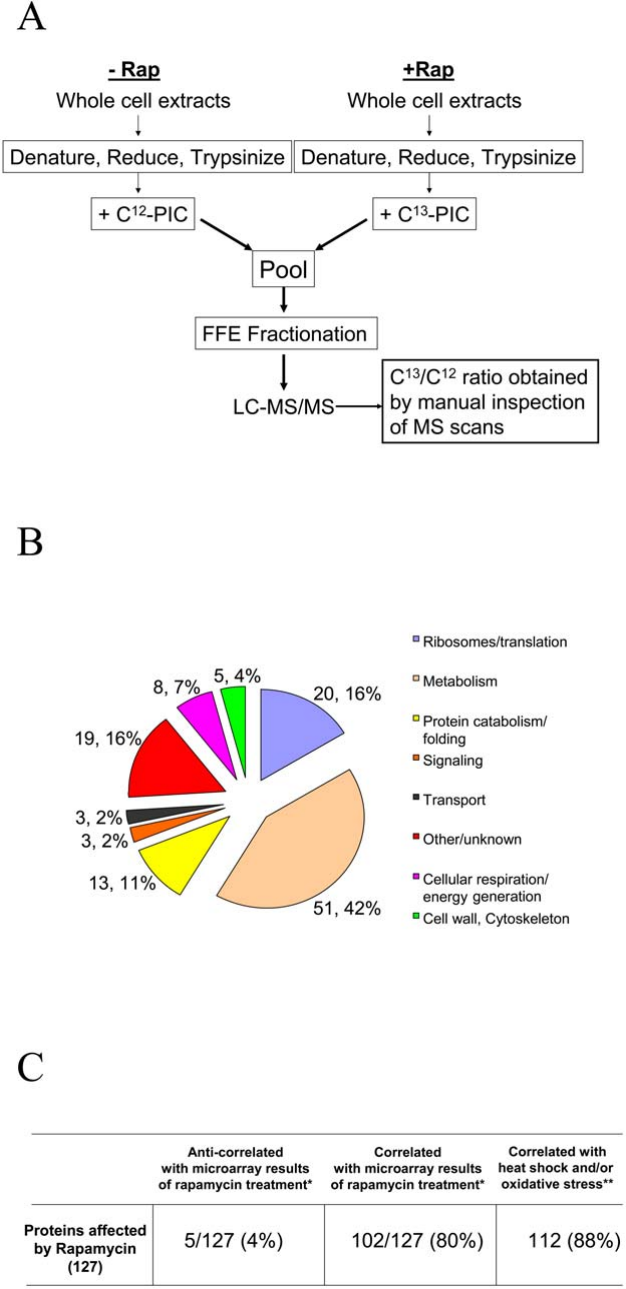
Proteomic analysis strategy and results. (A) Sample preparation workflow for quantitative proteomic analysis of rapamycin treatment in *BJ5465* yeast cells. (B) Functional categorization of 127 proteins showing abundance changes of 1.5 fold or greater due to rapamycin treatment. The number of proteins from each category, and their relative percentages are also indicated on the pie chart. (C) Correlation or anticorrelation (described as similar or opposite changes between proteins and RNA, respectively) for rapamycin affected proteins (obtained via proteomic analysis in this study) and gene transcripts (obtained by microarray analysis of rapamycin treated yeast cells; *[6], [7], and heatshock/oxidative stress; **[13]).

In this report, we describe results from a strategy combining quantitative proteomics and comparative expression analysis to obtain insights into TOR function and regulation in the budding yeast, *S. cerevisiae.* For the first step in this strategy, we profiled protein abundance changes in yeast cells treated with rapamycin, a highly specific inhibitor of Tor1/2 [18], [19]. **Figure 1A** outlines the quantitative proteomics method used. To limit protein degradation, the protease deficient strain, BJ5465 was used. Similar to other yeast strains [20]–[22], growth of BJ5465 slowed ∼70 minutes after treatment with 200 nM rapamycin (data not shown). Rapamycin-treated, and untreated cells were collected at this time point, and total protein isolated from each sample. 300 µg protein from each sample was digested with trypsin, and the separate peptide mixtures differentially labeled at their n-termini using ^12^C_6 _(‘light’) and ^13^C_6 _(‘heavy’) versions of phenyl isocyanate (PIC) [23]. Peptides from the rapamycin treated sample were labeled with ^13^C_6_-PIC, while those from the non-rapamycin treated sample (methanol alone) with ^12^C_6_-PIC (**Figure 1A**)_._ Combined samples were fractionated by preparative isoelectric focusing using Free Flow Electrophoresis (FFE) as described [24], and the peptide fractions analyzed by µLC-MS/MS on a linear ion trap instrument.

Using a combination of probability assignments and filtering based upon peptide pI, as we have previously described [24], we obtained a high confidence catalogue of 578 proteins (false positive rate <1%). Relative abundance ratios of each identified peptide, measured by the ratios of ^13^C to ^12^C signal intensities for each peptide, were obtained by manual examination of MS data. Based upon the average calculated abundance ratios across the entire protein catalogue, we determined that 1.5-fold and greater relative abundance changes were significant, as these ratios differed by over three standard deviations from the average protein ratio for the entire dataset. Using this significance threshold, 127 proteins (82 up-regulated, 45 down-regulated) representing a broad array of cellular functions showed changes in abundance. These proteins were grouped into functional categories using the ‘gene ontology’ function available on the yeast genome website (www.yeastgenome.org). The functional profile of rapamycin-affected proteins is shown in **Figure** **1B**, and supporting mass spectrometric data and quantitative information are shown for all proteins in supplementary information **(Table S1)**.

We first compared our proteomic dataset to data from previous microarray studies measuring transcriptional changes due to rapamycin treatment in yeast [6], [7]. We assumed that at least some of the abundance changes measured for specific proteins should be affected in a similar (i.e. homodirectional) manner in these studies. Among the 127 proteins which changed in abundance in our proteomic analysis, 102 also showed a homodirectional change in their corresponding mRNA transcripts (see **Figure 1C**). This high level of correlation between protein and mRNA behavior was observed in spite of the fact that microarray studies used for comparison were done using yeast from a different strain background (BY4741) than ours (BJ5465), and using different rapamycin treatment conditions (100 nM rapamycin either for 30 minutes [7] or over a time course up to 120 minutes [6]). This result may not be unexpected, however, given that rapamycin induced transcriptomic and translational state changes are positively coupled in yeast [21].

Almost half of the 45 total proteins showing a decrease in abundance in our dataset were either ribosomal proteins (RPs) or other translational components (See supplementary information, **Table** **S2**). This result is consistent with the well known role of TOR kinases in ribosomal biogenesis and protein translation [6], [20], [25]–[27]. With the exception of Pre10, Acs2, and Ppt1 (no mRNA expression data in presence of rapamycin is currently available for these), all of the proteins that decreased in abundance due to rapamycin treatment also showed decreased mRNA abundance in previous microarray analyses of rapamycin treatment [6], [7].

Consistent with the well-known role of TOR signaling in the regulation of metabolism, majority of proteins that increased in abundance upon rapamycin treatment fall into this general functional category (See **Table S2**). These proteins are involved in diverse aspects of metabolism, including amino-acid, carbohydrate, and nucleic acid metabolism. Several of these proteins regulate adaptation to poor nitrogen sources (proline, urea, allantoin) or carbon starvation. A majority of up-regulated proteins are also known to be affected at the mRNA level in a homodirectional manner (based on comparison with microarray data generated previously; [6], [7]).

Although the majority of the proteins show homodirectional changes with their mRNA transcripts upon rapamycin treatment, abundance changes of 17 proteins did not correlate with their mRNA transcripts. These proteins represent gene product responses to rapamycin treatment which could not have been predicted using microaray studies alone. Of these, five were actually anti-correlated (decreased in abundance at the mRNA level based on microarray experiments, but increased at the protein level; see **Figure 1C**). These anti- and non-correlated proteins and their magnitude of abundance increase were: Bmh1 (1.8 fold), Inh1 (2.2 fold), Qcr7 (1.6 fold), Ham1 (2.1 fold), Sbp1 (2.5 fold), Abf2 (27 fold), Crh1 (1.6 fold), Bgl2 (2.6 fold), Trr1 (1.9 fold), Pma1 (1.8 fold), Erv25 (1.6 fold), Cpr1 (1.7 fold), Pac10 (37 fold), YOL111C (3 fold), YLR301W (1.7 fold), Ppx1(52 fold), and Gvp36 (2.2 fold). Independent validation of these novel proteomic changes is necessary before experiments are designed based on these findings.

### Comparative expression analysis indicates a broad stress response due to rapamycin treatment

Although the analysis of our proteomics data above confirmed that our results were largely consistent with known effects of rapamycin treatment in yeast, it provided only limited insights into potential new pathways involved in regulation of TOR function. Therefore, as a next analysis step, we compared our proteomic profile of rapamycin treatment to existing expression data for corresponding gene products measured under a variety of conditions in yeast. We sought to identify conditions that resulted in similar proteomic or transcriptomic responses to those observed for rapamycin treatment, and use this information for obtaining insights into TOR regulation. Given that few datasets exist of proteomic changes due to systematic perturbation, even in a highly studied organism such as *S. cerevisiae*, we compared our dataset with currently available transcriptomic information from yeast exposed to a variety of environmental conditions [13]. This comparison was done ‘qualitatively’- looking for gene products which showed homodirectional changes (i.e. changed in the same direction) in our proteomic dataset and in microarray experiments, but not considering the magnitude of these changes in these different datasets.

Our comparative expression analysis revealed that 88% of proteins (112/127) showing an abundance change due to rapamycin treatment also showed homodirectional change at the mRNA level under conditions of heat/oxidative stress (**Figure 1C** **).** Based upon previous studies, the notion of TOR inhibition by rapamycin treatment activating a broad stress response in yeast is not surprising. Indeed, rapamycin treatment in yeast is known to induce a general stress response through the Msn2/4 transcription factor, resulting in increased transcription of its target genes [28], [29]. However, a closer look at our proteomic dataset showed that a number of the proteins affected by rapamycin treatment are not known targets of Msn2/4 [13], [30], [31]; these proteins also overlapped extensively between rapamycin and heat/oxidative stress (ribosomal proteins, for example). This suggested that involvement of additional regulatory factors might better explain the extent of overlap in affected genes under conditions of rapamycin treatment and heat/oxidative stress. At least some of the proteins showing abundance changes due to rapamycin treatment in our dataset are targets of other transcription factors that are known to be regulated by the TOR pathway in yeast [Gat1/Gln3, Rtg1/3, Crf1, Fhl1, and Spf1 [22], [25], [32], [33]]. However, little information exists to explain the similar abundance changes observed for their transcriptional outputs under conditions of rapamycin treatment and heat/oxidative stress. We also identified the stress regulator Hyr1 [34] in our proteomic analysis, which increased ∼17-fold (see **Table S2**), which could at least partially explain the extent of overlap between the two conditions. However, the targets regulated by Hyr1 in yeast are not extensively characterized, and thus its role in the observed overlap was not easily explained.

### Testing of the major regulators of stress response in yeast suggests a novel role for Hsf1 activation in inhibiting TOR/rapamycin resistance

The results of our comparative expression analysis suggested that existing information could not fully explain the extent of overlap in affected gene products under conditions of rapamycin treatment and heat/oxidative stress. This led us to investigate possible novel connections between stress regulators in yeast and the TOR pathway to better explain our observations. Specifically, we hypothesized that activation of regulator(s) of heat/oxidative stress response inhibits TOR function and/or signaling. To test our hypothesis, we investigated the effects of activation of the most well characterized, stress regulators in yeast, Msn2/4 [13], [35], [36], Hyr1 [34], and Hsf1 [37]–[39], on rapamycin resistance and TOR signaling.

Initially, we tested heat shock transcription factor 1 (Hsf1) for a possible role as a TOR inhibitor (for recent reviews on Hsf1, see [40], [41]). Hsf1 forms a homotrimer and recognizes heat shock elements (HSEs) in promoters of target genes consisting of at least three inverted repeats of nGAAn. Transcriptional targets of Hsf1 include molecular chaperones, heat shock proteins, and regulators of protein degradation/homeostasis, and are involved in regulating diverse signal transduction pathways as well as housekeeping functions within the cell [40]–[43].

To test for effects of Hsf1 activation on TOR signaling, we made use of mutants that are constitutively activated for Hsf1. One of the strains, *hsf1-R206S, F256S*, contains mutations in critical residues within the DNA-binding domain of *HSF1* [44]. The R206S and F256S substitutions are located in the ‘turn’ region and the fourth beta-sheet of the Hsf1 DNA-binding domain (DBD), respectively [45]–[47]. Importantly, these residues are not located in the third helix region of Hsf1 (which binds the nGAAn sequence), or in the trimerization domain of Hsf1, suggesting that these mutations would not affect sequence specificity of Hsf1 or its trimerization, respectively. The R206S substitution is expected to affect the DBD-DBD interaction, and F256S affects the activator function of Hsf1. The ability of *hsf1-R206S, F256S* cells to behave as a *HSF1* gain-of-function mutant is described in the next section. We also made us of *ssa1-3 ssa2-2* cells, which have been previously shown to be constitutively activated for Hsf1 because of the inability of mutated Ssa1/2 to autoregulate and inhibit Hsf1 function [48]-[50].

**Figure 2:**
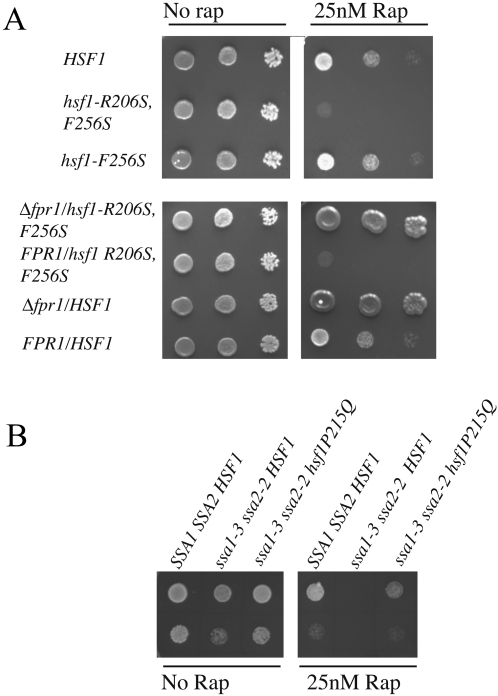
Cells with increased Hsf1 transcriptional activity are hypersensitive to rapamycin treatment. (A) Rapamycin sensitivity of *HSF1, hsf1-R206S, F256S*, and *hsf1-R256S* cells (upper panel). *FPR1*-dependent rapamycin sensitivity of *hsf1-R206S, F256S* cells (lower panel). (B) Rapamycin sensitivity of *SSA1 SSA2*, *ssa1-3 ssa2-2*, and *ssa1-3 ssa2-2 hsf1P215Q* cells. Cells were grown to saturation at 25°C and serial dilutions (50,000, 5000, and 500 cells per spot) were spotted on YPD plates supplemented with 25 nM rapamycin or drug carrier solvent (methanol) and assayed for growth at 25°C for the indicated durations of time. *ssa1-3 ssa2-2* cells and derivatives were grown identically but spotted at a density of 5000 and 500 cells/spot.

Consistent with reduced TOR signaling upon Hsf1 activation, *hsf1-R206S, F256S* cells were hypersensitive to rapamycin treatment at 25°C (**Figure 2A**). Cells with reduced TOR signaling are hypersensitive to rapamycin [14], [20]. In contrast, *hsf1-F256S* cells, a mutant with dysregulated Hsf1 function [47] were unaffected under the same conditions, indicating that dysregulation (i.e., qualitative change in function) of *HSF1* was not sufficient to cause rapamycin sensitivity (**Figure 2A** **, upper panel**). Furthermore, the rapamycin sensitivity of *hsf1-R206S, F256S* cells was completely suppressible by a deletion of the *FPR1* gene (**Figure 2A** **, lower panel**) indicating that these cells were hypersensitive to TOR inhibition specifically [18], [19]. Additionally, *hsf1-R206S, F256S* cells did not show sensitivity towards low doses of cycloheximide, arguing against a general drug-sensitivity of this mutant (data not shown).

As an independent means to assess the effect of Hsf1 activation on rapamycin sensitivity, we also assayed *ssa1-3 ssa2-2* cells for growth in the presence of rapamycin. As shown in **Figure 2B**, these cells were also hypersensitive to rapamycin treatment at 25°C. Importantly, decreasing Hsf1 function in these cells by an *hsf1P215Q* mutation [49], [51] suppressed their rapamycin sensitivity significantly, demonstrating that the rapamycin sensitivity of *ssa1-3 ssa2-2* cells was dependent on Hsf1 activation. In contrast to our observations in cells with constitutively active Hsf1, hypomorphic or dysregulated alleles of *hsf1* (*HSF1/hsf1Δ, hsf1-ba1, hsf1-AR1, hsf1-N583, or hsf1-F256S;* [47], [52]–[54]) were essentially unaffected for rapamycin resistance (data not shown), suggesting the basal function of Hsf1 or its dysregulation does not affect rapamycin resistance/TOR signaling in *S. cerevisiae*.

### hsf1-R206S, F256S mutant cells have increased activity of Hsf1 in a temperature-sensitive manner

**Figure 3:**
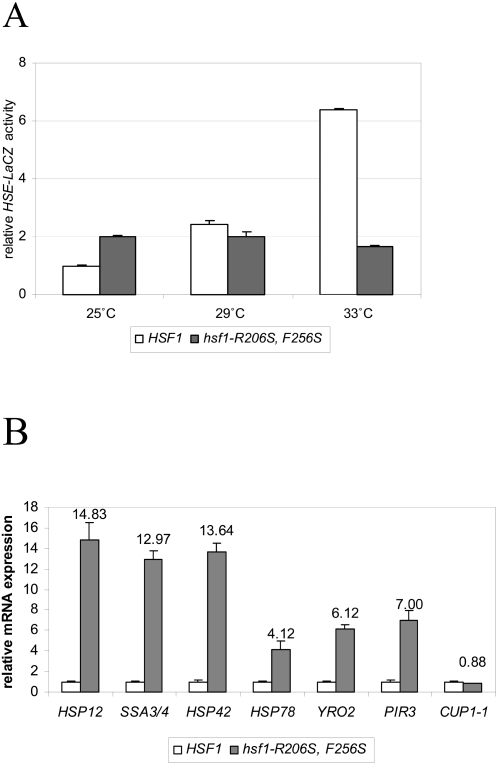
Effect of *hsf1-R206S, F256S* mutation on expression of HSE4Ptt-*CYC1*-*LacZ* reporter and Hsf1 target genes. (A) *hsf1-R206S, F256S* and isogenic *HSF1* cells transformed with HSE4Ptt-*CYC1-lacZ* plasmid [53] were grown overnight in minimal selective media at 23°C to an OD_600_ of 0.5 units, and then shifted to 25°C, 29°C, or 33°C, for 90 minutes prior to determination of β-galactosidase activity. (B) mRNA levels of diverse classes of Hsf1 targets in *hsf1-R206S, F256S* cells relative to *HSF1* cells. The promoter region of *HSP12* is known to have ‘step’ heat shock elements (HSEs), while that of *SSA3/4, HSP78*, and *HSP42* have perfect HSEs [44]. Although canonical HSEs have not been found in promoter regions of *PIR3* and *YRO2*, these were identified in global CHIP-on-CHIP experiments as Hsf1 targets [55]. *CUP1-1* has a variant HSE [44]. Cells were grown at 25°C, and processed for RNA isolation, real-time PCR analysis, and analyzed as described in materials and methods section. Relative expression of each gene was normalized to actin and expressed as an average fold induction in *hsf1-R206S, F256S* cells versus unperturbed wild type cells.

We further investigated the effect of the *R206S, F256S* mutation on Hsf1 activity at a variety of temperatures. The *hsf1-R206S, F256S* mutation has been recently demonstrated to have a severe defect in the expression of multiple Hsf1 targets under heat shock conditions [44]. Consistent with this result, these cells displayed dramatically reduced transcriptional activity at 33°C against the HSE4Ptt-*CYC1-LacZ* reporter (**Figure 3A**). However, at 29°C, their activity was roughly comparable to wild type cells and at 25°C, *hsf1-R206S, F256S* cells had a 2-fold increase in transcriptional activity (**Figure 3A**). Thus, *hsf1-R206S, F256S* cells have enhanced basal activation of Hsf1 at 25°C towards a synthetic reporter of Hsf1 activity.

To further confirm that *hsf1-R206S, F256S* cells had enhanced basal activation of Hsf1 at 25°C, we tested the expression level of numerous known transcriptional targets of Hsf1 in these cells. Hsf1 targets have been classified into those that contain either ‘perfect’ heat shock elements (HSEs) or those that contain discontinuous heat shock elements (‘gap’ type and ‘step’ type) [44]. As shown in **Figure 3B**, Hsf1 targets with ‘step’ type HSEs (*HSP12*), or perfect HSEs (*SSA3/4, HSP42*, and *HSP78*), were increased dramatically for expression in these cells at 25°C compared to wild-type, whereas *CUP1-1* (which has ‘gap’ type HSEs) was nearly unaffected for expression under these conditions. Hsf1 targets without consensus heat shock elements in their promoter elements (identified by global CHIP-on-CHIP analysis [55]), such as *PIR3*, and *YRO2*, were also increased in expression in these cells. These results led us to conclude that *hsf1-R206S, F256S* cells largely behave as an *hsf1* gain-of-function mutant at 25°C. Our observation is in agreement with previous reports demonstrating that mutation of the same residues in Hsf1 with different amino acid substitutions (*R206S, F256* ***Y*** *vs. R206S, F256* ***S***) also enhanced basal transcriptional activity of Hsf1 2–3 fold (using a synthetic reporter of Hsf1 activity [56]). Consistent with our findings for the *hsf1-R206S, F256S* cells, *hsf1-R206S, F256Y* cells were also found to be hypersensitive to rapamycin treatment (data not shown).

### hsf1-R206S, F256S cells display reduced TOR signaling

Given our results showing *FPR1*-dependent rapamycin sensitivity of *hsf1-R206S, F256S* cells, we tested for effects on Tor1/2 protein levels and TOR signaling. We found that *hsf1-R206S, F256S* cells did not show decreased Tor1 and Tor2 protein levels compared to wild type cells, as assessed by western blotting (data not shown). Hence, we tested for effects on TOR signaling in *hsf1-R206S, F256S* cells.

**Figure 4:**
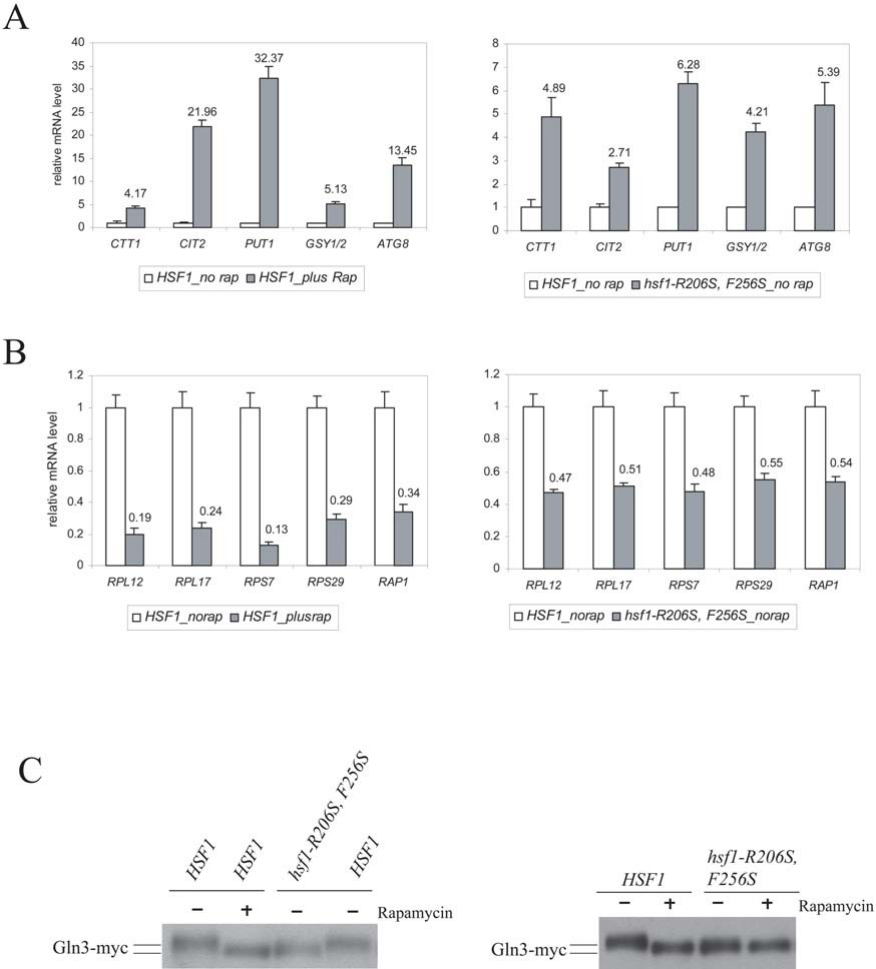
Reduced TOR signaling in *hsf1-R206S, F256S* cells. (A) Expression level of genes representing five different pathways repressed by TOR function, upon rapamycin treatment in *HSF1* cells (left panel), and in *hsf1-R206S, F256S* cells (right panel, in absence of rapamycin treatment). (B) Expression level of ribosomal protein (RP) genes and *RAP1*, a positive regulator of RP genes, upon rapamycin treatment in *HSF1* cells (left panel) and in *hsf1-R206S, F256S* cells (right panel, in absence of rapamycin treatment) (C) Mobility of Gln3-*myc*_13_ in *HSF1* cells treated with or without rapamycin and *hsf1-R206S, F256S* cells with or without rapamycin treatment as indicated above. Cells were grown to log-phase at 25°C and treated with 200nM rapamycin or methanol alone and processed for RNA isolation or total protein extraction as described in materials and methods section.

In yeast, activated Tor1/2 complex inhibits the expression of genes involved in stress pathways, autophagy, metabolite accumulation (glycogen synthesis), retrograde signaling and Nitrogen Catabolite Repression (NCR) pathways, while it promotes expression of ribosomal protein (RP) genes as well and their positive regulators ([1]–[3] and references therein). We utilized quantitative real-time PCR to monitor expression levels of representative genes of each of these TORC1-regulated pathways as an initial ‘readout’ of TOR signaling. As expected, rapamycin treatment in *HSF1* cells, caused elevated expression of genes from each of the TOR-inhibited pathways, and reduced expression of ribosomal protein (RP genes) (see **Figures 4A and 4B**, left panels).

Consistent with reduced TOR signaling, *hsf1-R206S, F256S* cells exhibited elevated expression of genes from each of the TOR-inhibited pathways (see **Figure 4A**, right panel). The affected genes include the Msn2/4 target gene, *CTT1* [36], [57] (increased 4.9-fold), the NCR gene, *PUT1* [58], [59] (increased 6.3 fold) and the Rtg1/2 target gene, *CIT2* [58], [59] (increased 2.7-fold). Additionally, the regulator of the last step in glycogen synthesis, *GSY1/2*, known to be induced upon TOR inhibition [20], [28], [60], increased 4.2-fold. The autophagic marker Atg8/Aut7 [61], increased 5.4-fold. Also, we found reduced expression of ribosomal protein genes and their positive regulators, such as *RAP1*, in *hsf1-R206S, F256S* cells (see **Figure 4B,** right panel). Thus the expression profile of multiple TOR-regulated genes is consistent with reduced TOR signaling in *hsf1-R206S, F256S* cells.

As further evidence for reduced TORC1 function in *hsf1-R206S, F256S* cells, we assayed Gln3p mobility/phosphorylation, since this represents a direct physiological substrate of the TOR kinase in yeast cells [29], [62]. TOR kinase activity promotes phosphorylation of Gln3, while rapamycin treatment results in its dephosphorylation. De-phosphorylated Gln3p runs faster on an SDS-PAGE gel compared to its phosphorylated counterpart ([29], [62], **Figure 4C**). Consistent with reduced Gln3 phosphorylation (and reduced TOR function), Gln3-*myc*_13_p runs faster in *hsf1-R206S, F256S* cells compared to *HSF1* cells (see **Figure 4C**, left panel). Mobility of this faster migrating form of Gln3-*myc*_13_p is enhanced further by rapamycin treatment in *hsf1-R206S, F256S* cells suggesting an intermediate effect on Gln3 phosphorylation (when compared to rapamycin treatment, **Figure 4C**, right panel). This result is in good agreement with the expression analysis of TORC1 regulated genes (See **Figures 4A and 4B**) which also showed a less dramatic effect on TOR functional ‘readouts’ in *hsf1-R206S, F256S* cells than rapamycin treatment of *HSF1* cells.

### Msn2/4 and Gln3 are necessary for full induction of TOR-repressed genes in hsf1-R206S, F256S cells

Inhibiting TORC1 function (by rapamycin treatment for example) causes nuclear localization/activation of multiple transcription factors, including Msn2/4, and Gat1/Gln3, and elevated expression of their target genes [22], [29], [32], [63]. Thus, if *hsf1-R206S, F256S* cells have reduced TOR function, then the elevated expression of TORC1-inhibited genes (some of which are shown in **Figure 4A**) should be dependent on Msn2/4 and Gat1/Gln3. To test this hypothesis, we analyzed effects of their deletion in *hsf1-R206S, F256S* cells.

**Figure 5:**
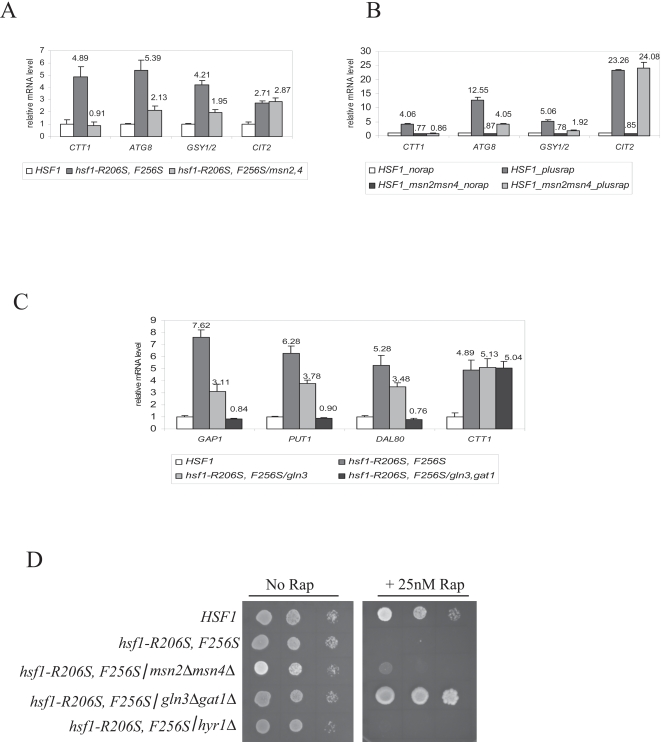
Role of Msn2/4 and Gln3/Gat1 in TOR-regulated phenotypes seen in *hsf1-R206S, F256S* cells. (A) Effect of deleting *MSN2, 4* on elevated expression of Msn2/4 targets in *hsf1-R206S, F256S* cells (B) Effect of deleting *MSN2, 4* on rapamycin induced expression of Msn2/4 targets in *HSF1* cells (C) Effect of deleting *GLN3* alone or both *GLN3* and *GAT1* on elevated expression of NCR genes in *hsf1-R206S, F256S* cells (D) Effect of *MSN2/4*, *GLN3/GAT1*, or *HYR1* deletions on rapamycin sensitivity of *hsf1-R206S, F256S* cells. Relative expression of each gene was normalized to actin and expressed as an average fold induction relative to wild type cells.

Upon deletion of *MSN2* and *MSN4*, elevated expression of its target genes *CTT1, GSY1/2* and *ATG8* (all of which have Msn2/4 binding sites in their promoter elements), but not *CIT2* (target of Rtg1/3), was reduced in *hsf1-R206S, F256S* cells (see **Figure 5A**). Elevated expression of *CTT1* in particular, was completely abolished. Although *MSN2,4* deletion suppresses expression of *GSY1/2* and *ATG8* only partially, this likely does not indicate a direct activating effect of the variant *hsf1-R206S, F256S* protein on Msn2,4 target genes, as similar results were also observed in rapamycin treated *HSF1 msn2Δmsn4Δ* cells (see **Figure 5B**). As shown in **Figure 5C**, deletion of both *GLN3* and *GAT1* abrogated expression of multiple NCR genes (*GAP1, PUT1, DAL80*), but not *CTT1* (which is Msn2/4 dependent instead), in *hsf1-R206S, F256S* cells (see **Figure 5C**). Furthermore, combining *hsf1-R206S, F256S* cells with *msn2Δmsn4Δ* or *gln3Δgat1Δ* suppresses the rapamycin sensitivity of *hsf1-R206S, F256S* cells; however, the effect of *msn2Δmsn4Δ* is very modest when compared to *gln3Δgat1Δ* (see **Figure 5D**). Taken together, these results provide genetic evidence for activation of TORC1-inhibited transcription factors in *hsf1-R206S, F256S* cells.

### Elevated expression of PIR3 and YRO2 inhibits rapamycin resistance and TOR signaling in hsf1-R206S, F256S cells

**Figure 6:**
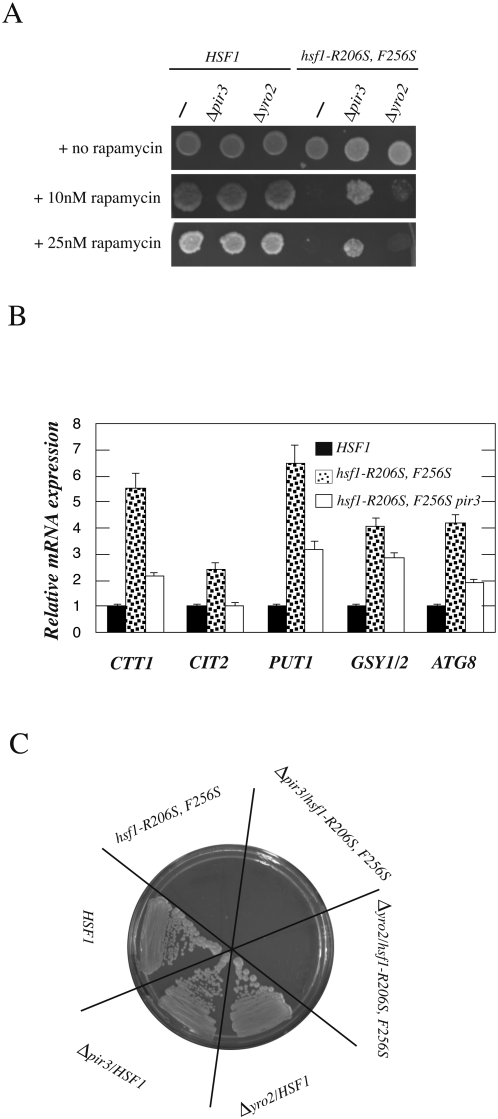
Deletion of Hsf1 target genes, *PIR3* and *YRO2* partially suppress TOR-regulated phenotypes of *hsf1-R206S, F256S* cells. A) Suppression of rapamycin sensitivity of *hsf1-R206S, F256S* cells by deletion of *PIR3* and *YRO2*. *HSF1* and *hsf1-R206S, F256S* cells bearing the indicated gene deletions were grown to saturation at 25°C and 5000 cells each were spotted on YPD plates supplemented with methanol alone (rapamycin solvent), 10 nM, and 25 nM rapamycin, respectively. B) *PIR3* deletion reduced expression of multiple TOR-repressed genes in *hsf1-R206S, F256S* cells. Expression level of genes was monitored by RT-PCR as explained in materials and methods section. C) Effect of *PIR3* and *YRO2* deletion on temperature sensitivity of *hsf1-R206S, F256S* cells. Indicated strains were streaked out on YPD plates and allowed to grow 3 days at 34°C.

To explain the observed effects on TOR-regulated signaling in *hsf1-R206S, F256S* cells, we considered the possibility that elevated expression of select Hsf1 targets might contribute to these phenotypes. A number of Hsf1 target genes that were elevated for expression in these cells (*HSP12, HSP30, HSP42, HSP78, SSA4, HSP104, PIR3* and *YRO2,* see **Figure 3B**) were deleted in *hsf1-R206S, F256S* cells, and tested for effects on rapamycin sensitivity. Most of the deletions had essentially no effect on the rapamycin sensitivity of *hsf1-R206S, F256S* cells (data not shown). However, as shown in **Figure 6A**, deletion of *YRO2* partially suppressed the rapamycin sensitivity of *hsf1-R206S, F256S* cells at 10 nM rapamycin, and deletion of *PIR3* suppressed strongly the rapamycin sensitivity of these cells at both 10 nM and 25 nM rapamycin. Importantly, deletion of these genes had no effect on the rapamycin sensitivity of wild type cells, indicating that their basal expression level did not inhibit rapamycin resistance.

*PIR3* is a heat inducible, glycosylated protein that is a structural component of the yeast cell wall, and required for tolerance of yeast to heat shock and osmotin [13], [64], [65]. *YRO2* is a gene of unknown function that is also heat inducible, localized to the cell periphery and bud, in particular to the cell membrane and mitochondria [13], [66]–[68]. Although neither of these genes have well defined heat shock elements in their promoter regions, these genes were previously identified as Hsf1 targets in a global CHIP-on-CHIP analysis [55].

Given that *PIR3* was a strong suppressor of the rapamycin sensitivity of *hsf1-R206S, F256S* cells, we tested if its deletion might also augment TOR signaling in these cells. Supporting this notion, expression levels of diverse TOR-inhibited genes (*CTT1, CIT2, PUT1, GSY1/2*, and *ATG8;* see **Figure 6B**), was each reduced upon *PIR3* deletion in *hsf1-R206S, F256S* cells. Additionally, expression of multiple RP genes was also augmented partially in *hsf1-R206S, F256S* cells by *PIR3* deletion (see supplementary information; **Figure S1**). *PIR3* or *YRO2* deletion did not suppress the temperature-sensitivity of *hsf1-R206S, F256S* cells indicating specificity towards TOR-related phenotypes of these cells (**Figure 6C**). Taken together, these results demonstrate that elevated expression of specific Hsf1 target genes inhibits rapamycin resistance and TOR signaling in *hsf1-R206S, F256S* cells.

### Consitutive activation of Msn2/4 or Hyr1 does not inhibit TOR signaling

Having shown that cells with constitutively active Hsf1 display reduced TOR signaling, we then asked if cells activated for additional heat/oxidative stress induced transcription factors also displayed similar phenotypes (to test if this observation was unique to Hsf1). Towards this aim, we tested if overexpression of *MSN2*, *MSN4* or *HYR1* might also inhibit TOR signaling (similar to what was seen upon *HSF1* activation). Overexpression of each of these genes was achieved by 2μ plasmids previously used by others [69], [70] and verified by real-time PCR (data not shown).

**Figure 7:**
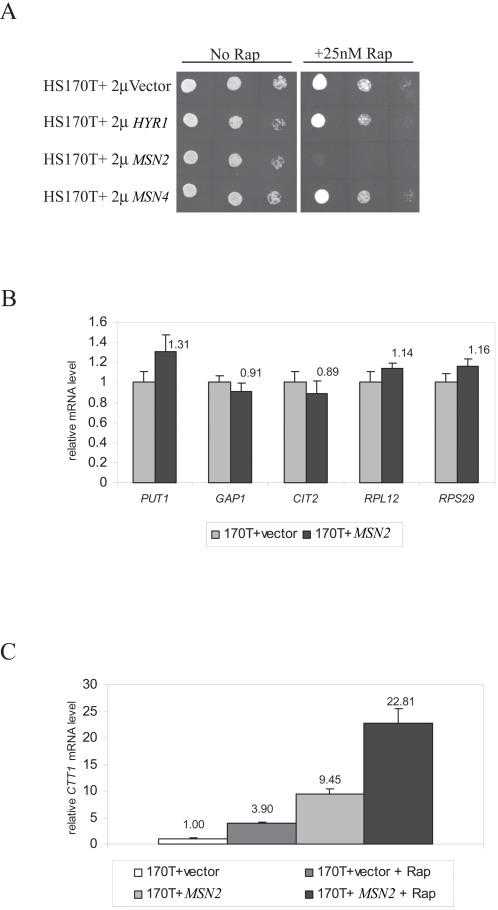
Over expression of *MSN2*, *MSN4* or *HYR1* does not inhibit TOR signaling (A) Effect of over expression of *MSN2*, *MSN4* or *HYR1* on rapamycin resistance of wild type cells. Wild type HS170T cells (*HSF1* cells isogenic to *hsf1-R206S, F256S* cells used in this study) were transformed with 2μ plasmids for over expression of the relevant genes, and spotted on selective media supplemented with 25 nM Rapamycin (or methanol) at 50,000, 5000, and 500 cells per spot and assayed for growth at 25°C (B) Effect of *MSN2* over expression on TOR signaling ‘readouts’ assayed by real-time PCR (C) Effect of *MSN2* over expression versus rapamycin treatment, on expression level of *CTT1*, a classical Msn2 target gene. RNA isolation, cDNA synthesis, real-time PCR conditions, and analysis of data are described in materials and methods section.

As shown in **Figure 7A**, overexpression of *MSN4* or *HYR1* was not sufficient to cause rapamycin sensitivity, arguing against the notion that these genes could act as putative TOR inhibitors. Interestingly, *MSN2* overexpression did confer rapamycin sensitivity (**Figure 7A**). However, this sensitivity was not accompanied by attenuated TOR signaling as assessed by expression analysis of TORC1-regulated genes (See **Figure 7B**). These results point instead to the possibility that overexpression of Msn2 targets inhibits rapamycin sensitivity due to elevated expression of some of its target genes, and that these do not inhibit TOR signaling akin to Hsf1 target genes. Indeed, *MSN2* overexpression caused a dramatic increase in expression of its target gene, *CTT1* (when compared to the increase due to rapamycin treatment, see **Figure 7C**). Collectively, these results further support a novel role for activated Hsf1 among the stress activated transcription factors in putatively inhibiting TOR signaling via elevated expression of its target genes.

## Discussion

In this study, we have performed the first proteomic profiling of rapamycin treatment in *S. cerevisiae,* and used this information for comparative expression analysis with existing expression data measured under different conditions. Our aim was to use this information for identifying novel relationships between regulators of known biological pathways and TOR function. Additionally, we also sought to identify protein abundance changes that could not be predicted from previous microarray analyses of rapamycin treatment [6], [7] to gain new insights into TOR function. Although the total number of proteins identified with high confidence (578) was relatively small compared to other proteomic studies in yeast, (most likely due to the charge-neutralizing effect on peptide n-termini of the PIC label incorporated for quantitative analysis [23]), we were able to identify abundance changes for 127 proteins upon rapamycin treatment. Among these, 17 proteins were found increased in abundance upon rapamycin treatment that do not show similar changes in their corresponding mRNA transcripts. Among these, increased abundance of Ppx1 and Inh1 upon rapamycin treatment is of particular interest, since Ppx1 overexpression inhibited mTOR activity in mammalian cells [71], whereas *inh1Δ* cells were reportedly rapamycin resistant [16]. Our proteomic findings thus suggest that the induction of these proteins might potentiate TOR inhibition and promote rapamycin sensitivity in yeast, although further study is necessary to confirm this possibility. 

Using comparative expression analysis of our proteomic dataset and existing microarray gene expression data, we observed extensive overlap in gene products affected by rapamycin treatment and conditions of heat/oxidative stress. Although the activation of stress genes by rapamycin treatment has been noted by other groups previously, it has been attributed mostly to the activation of Msn2/4 under these conditions [6], [29]. However, a majority of the affected proteins we identified are not known to be regulated by Msn2/4. Additionally, little information currently exists about the other known downstream responses of TOR inhibition to explain the extent of overlap observed between rapamycin treatment and heat/oxidative stress. Preiss et al [21] have demonstrated that rapamycin and heat shock induced changes in the transcriptome are amplified at the translational level. However, to the best of our knowledge a direct comparison of the specific genes affected under each of these conditions, as done here has not been reported previously.

Based upon the results of our comparative expression analysis, we hypothesized that the activation of a regulator(s) of heat shock/oxidative stress response inhibits TOR function and/or signaling. Because these stress responses in yeast are controlled by three main transcription factors, Msn2/4 [13], [35], [36], Hyr1 [34], and Hsf1 [37]–[39], we explicitly tested for a putative role of their activation in the inhibition of TOR signaling and rapamycin resistance. Unlike other transcription factors tested, Hsf1 is unique since cells constitutively activated for Hsf1 (*hsf1-R206S, F256S* cells) specifically display multiple phenotypes consistent with reduced TOR function. Several lines of evidence support this conclusion. First, genes representing five different biological functions (Stress genes, RTG signaling, NCR genes, Glycogen synthesis, and Autophagy) which are inhibited by Tor1/2 in yeast, are all elevated for expression in *hsf1-R206S, F256S* cells. Second, multiple ribosomal protein genes (which are known to be down-regulated upon TOR inhibition) are also reduced for expression in *hsf1-R206S, F256S* cells. Third, western blotting indicates a faster migrating form of Gln3p in these cells, consistent with reduced phosphorylation of this physiological substrate of TORC1. Fourth, genetic data support that the TORC1 inhibited transcription factors, Msn2/4 and Gln3/Gat1 are activated in *hsf1-R206S, F256S* cells. Finally, *hsf1-R206S, F256S* cells are hypersensitive to rapamycin treatment in an *FPR1*-dependent manner, indicating sensitivity to TOR inhibition.

Elevated expression of specific Hsf1 target genes in *hsf1-R206S, F256S* cells contributes to the TOR-regulated phenotypes seen in these cells. This conclusion is based on our finding that deletion of *PIR3* and *YRO2* suppresses rapamycin sensitivity and *PIR3* deletion also augments TOR signaling in *hsf1-R206S, F256S* cells. In contrast, their deletion has no effect in wild-type cells (where their expression is baseline compared to *hsf1-R206S, F256S* cells). This also explains why *PIR3* or *YRO2* have not previously been identified in global screens of rapamcyin fitness in yeast [14]–[16]. Also, neither of these genes have been identified in studies using galactose-inducible overexpression of yeast genes to identify regulators of rapamycin resistance [17]. Potential reasons for this include the possibility that galactose-inducible library used by this group did not express *PIR3* and *YRO2*, or that their overexpression does not inhibit rapamycin resistance on alternative carbon sources such as galactose, or that they act in concert with other Hsf1 target genes to affect TOR signaling and rapamycin resistance. Finally, hypomorphic or dysregulated alleles of *hsf1* were unaffected for rapamycin resistance, further supporting a role for Hsf1 activation induced targets specifically in inhibiting yeast TOR.

Additional work is necessary to determine the mechanism(s) by which Hsf1 activation and the resultant elevated expression of *PIR3* and *YRO2* putatively impinge on the TOR pathway. The cell wall localization of Pir3 and integral membrane localization of the 7-membrane protein, Yro2, places them in proximity to the TOR kinases which are membrane associated themselves [72], [73]. It is noteworthy that both TOR and Hsf1 function have been previously implicated as being involved in aspects of cell wall integrity via effects on the PKC/Mpk1 cascade [54], [74], [75], and deletion of genes affecting cell wall integrity can affect rapamycin resistance, and potentially TOR [16]. We found that several putative rapamycin protective genes, were decreased for expression in *hsf1-R206S, F256S* cells; however, there was no effect of *PIR3* and *YRO2* deletions on the reduced expression level of these putative TOR regulators in *hsf1-R206S, F256S* cells (data not shown). Thus, alterations in their expression levels are unlikely to represent the basis of *PIR3/YRO2* mediated effects in *hsf1-R206S, F256S* cells.

In yeast, TOR signaling has been shown to bifurcate into at least two distinct effector pathways regulated by Tap42/Sit4 and Ras/cAMP/PKA [28]. While the former affects NCR gene expression via Gln3/Gat1 activation, the latter regulates the effect of the TOR pathway on RP gene expression and Msn2/4 activation. We have found that *hsf1-R206S, F256S* cells are affected in both of these effector branches of TOR signaling, and that *PIR3* deletion suppresses ‘readouts’ of both effector branches. Thus, we propose that Hsf1 activation and its target gene products putatively act upstream of these TOR signaling effectors. However, we cannot formally rule out the possibility that Hsf1 activation might also act parallel to the TOR pathway. Additional targets of Hsf1 might play a role in this regulation as well. Further work is necessary using a combination of genetic and transcriptomic or proteomic analyses to identify the entire spectrum of Hsf1 targets involved, and determine their connections with the known upstream regulators of the TOR pathway in yeast.

We have also tested for the effect of TOR inhibition on Hsf1 transcriptional activity. Cells expressing a plasmid borne synthetic reporter of Hsf1 transcriptional activity (HSE-4Ptt-*CYC1-LacZ*) were unaffected for *LacZ* expression either upon deletion of *TOR1* or treatment with various concentrations of rapamycin (data not shown). Additionally, only about 10% of the 165 known direct targets of Hsf1 [55] are induced in microarray analyses of rapamycin treatment, arguing against a general activation of Hsf1 [6], [7]. Thus, unlike the stress regulators Msn2/4 and Hyr1, TOR inhibition does not activate Hsf1 under these conditions. Consistent with these results, dietary restriction (which can cause TOR inhibition) in *C. elegans* does not significantly activate expression from a reporter of Hsf1 activity (hsp-16.2:GFP, for example) [76], [77]. Rather, our results are consistent with Hsf1 activation inhibiting TOR signaling in yeast.

It would be interesting to test if a similar relationship between Hsf1 and the TOR pathway existed in higher organisms as well. Supporting such a possibility, activation of Hsf1 or TOR inhibition promote lifespan in *C. elegans* [76], [78], [79]. However, the effects of TOR depletion are independent of DAF-16 in *C.elegans* (unlike that of HSF-1 activation), raising doubt on the possibility that Hsf1 activation promotes lifespan via a putative inhibitory effect on the TOR pathway. Hsf1 activation or TOR inhibition cause clearance of aggregation-prone proteins in higher organisms [80]–[82], but it remains unknown if potential connections between Hsf1 activation and mTOR exist and contribute to these phenotypes. Arguing against such a possibility, we have found that Celasterol treatment of Hela cells, (Celasterol causes pharmacological activation of Hsf1 via an unknown mechanism [83]), did not cause reduction in phosphorylation of the mTOR subtrate, S6K protein (Bandhakavi S and Griffin TJ., unpublished results). Future studies will shed further light on the possible conservation of yeast Hsf1/TOR relationship in other organisms.

In conclusion, our findings provide intriguing new insights into the relationship between stress signals and cellular growth inhibition. Additionally, our results highlight the value of performing comparative expression analysis between proteomic and genomic datasets to reveal new regulatory connections. Comparative expression analysis is often used in microarray-based analyses of expression changes due to systematic perturbation to find overlapping effects on biological pathways. However, it is usually not an option in quantitative proteomic profiling based studies because of the paucity of protein expression data obtained under various experimental conditions. Our results show that a qualitative comparison of proteomic and transcriptomic datasets, looking for homodirectional changes between among gene products common to these datasets, has value in identifying novel regulatory connections. Such an approach takes advantage of the wealth of microarray based studies that are currently available and can therefore be a useful tool for enhancing the information gained from proteomic profiling studies.

## Materials and Methods

### Strains and growth conditions

The protease deficient strain BJ5465 (*MATa ura3-52 trp1 leu2-delta1 his3-delta200 pep4::HIS3 prb1-delta1.6R can1 GAL*) was obtained from ATCC, and used for protein extraction following rapamycin treatment. Cells expressing wild type or mutant *HSF1* (*HSF1, hsf1-R206S,F256S, hsf1-ba1, hsf1-AR1Δ, hsf1-N583,* and *hsf1-R256S)* and the isogenic version of *msn2Δmsn4Δ* were obtained from Dr. Hiroshi Sakurai (Kanazawa University, Japan). *hsf1-R206S, F256Y* cells and isogenic *HSF1* cells were generously gifted by Dr. Dennis Winge (University of Utah Health Sciences Center, Salt Lake City, UT). *ssa1-3 ssa2-2, ssa1-3 ssa2-2 hsf1P215Q* and isogenic wild type cells were obtained from Dr. Elizabeth Craig (University of Wisconsin, Madison, WI). *HSF1/HSF1* and *HSF1/hsf1Δ* cells were obtained from Open Biosystems (www.openbiosystems.com). *FPR1, HYR1, GLN3* or Hsf1 target gene deletions were made by PCR generation of a homology cassette using a *KanMX6* resistance module as a dominant marker. *GAT1* deletion was made using *URA3* as a dominant marker. All disruptions were confirmed by PCR. To combine deletion of *msn2Δmsn4Δ* with *hsf1-R206S, F256S* cells, *YCP-TRP1-hsf1-R206S, F256S* plasmid was transformed into *msn2Δmsn4Δ* cells and the wild type *HSF1* plasmid was shuffled out using 5-FoA. Sensitivity to rapamycin was determined by spotting serial dilutions of wild type and mutant strains on minimal media or YPD plates supplemented with rapamycin (dissolved in methanol) to a final concentration of 10 nM or 25 nM. Media supplemented with methanol alone were used for plates without rapamycin. Sensitivity to cycloheximide was carried out identically on YPD plates supplemented with cycloheximide at 0.025 µg/ml concentration in DMSO or DMSO alone.

### Sample preparation for proteomic analysis

BJ5465 cells were grown in liquid YPD that was either supplemented with rapamycin at a final concentration of 200 nM (dissolved in methanol), or methanol alone. 70 minutes into drug treatment, cells were collected and proteins extracted by boiling in SDS sample buffer followed by vortexing in presence of glass beads [84], [85]. Extracted proteins were precipitated by TCA, dissolved in 50mM Tris, 1%SDS, 5mM EDTA, and exchanged into 50mM Hepes-KOH, pH7.5. 300 µg of protein from rapamycin treated or control sample was trypsinized overnight and labeled with ^13^C_6_- or ^12^C_6_-versions of phenyl isocyanate (PIC) essentially as described previously [23].

### Peptide fractionationation and mass spectrometric analysis

After labeling, samples were pooled, desalted and concentrated using a mixed mode cation exchange (MCX) cartridge (Waters), and fractionated by preparative isoelectric focusing using a Free Flow Electrophoresis (FFE, BD Biosciences, Inc.) as described [24]. Immediately after FFE fractionation, the pH in each well of the microtiter plate was measured using a micro pH electrode. Peptides were resolved over a pH range of ∼3–10. 10% of the sample was removed from each well across the pH gradient, and subjected to ultrafiltration to remove contaminating high molecular weight HPMC polymer components of the ampholyte mixtures. The filtrate was dried under vacuum and then loaded to a microcapillary reverse-phase liquid chromatography (µLC) column and analyzed online by automated tandem mass spectrometry (MS/MS) using a Thermo-Fisher LTQ two-dimensional linear ion trap instrument. Samples were automatically loaded across a Paradigm Platinum Peptide Nanotrap (Michrom) pre-column (0.15 x 50 mm, 400 µl volume) for sample concentrating and desalting, at a flow-rate of 50 µl/min in HPLC buffer A prior to loading into an inline analytical capillary column (75 µm x 12 cm) with C18 resin (5 µm, 200A° Magic C18AG, Michrom) and Picofrit capillary tubing (New Objective, Cambridge, MA). Peptides were eluted using a linear gradient of 10–35% buffer B over 60 minutes, followed by isocratic elution at 80% buffer B for 5 minutes with a flow rate of 0.25 µl/min across the column. The electrospray voltage was set to 2.0 kV. A data-dependent acquisition method was employed, in which each full scan was followed by a high resolution zoom scan of each precursor peptide mass prior to MS/MS analysis, in order to provide more accurate quantitative measurements of PIC labeled peptide pairs. The four most intense precursor ions from each full scan were selected for MS/MS. Selected precursor masses were excluded from selection for MS/MS for 30 seconds. Each full scan consisted of 1 microscan with a maximum fill time of 50 milliseconds; each MS/MS scan consisted of 1 microscan with a maximum fill time of 100 milliseconds.

### Sequence Database Searching and Data analysis

All MS/MS data was analyzed by sequence database searching using the program Sequest [86] against protein sequences derived from all known open reading frames in *S. cerevisiae.* In order to distinguish correct peptide matches from incorrect matches, we used a combination of probability scores using the probabilistic scoring algorithm, Peptide Prophet [87], and the difference between predicted and observed isoelectric points of PIC labeled peptides, essentially as described previously [24]. The charge on the N-terminus of peptides was set to zero in theoretical pI calculations due to the addition of the uncharged PIC group [23]. False positive rate of identification was estimated as described before [88]. After pI filtering, a threshold Peptide Prophet Probability score of 0.47 for peptide matches was used, providing an estimated false positive rate of 1%. Full scan mass spectra of peptide sequence matches were inspected, the relative intensities of light and heavy labeled peptide pairs measured, and relative abundance ratios calculated (shown as C13/C12 ratios in Supplementary Tables S1 and S2).

### RNA isolation, real-time PCR analysis

*HSF1*, *hsf1-R206S, F256S* cells and strain derivatives were grown in liquid YPD/minimal media at 25°C or 29°C to log phase prior to treatment with 200 nM rapamycin for 30 minutes. Total cellular RNA was isolated using the Masterpure yeast RNA purification kit (Epicentre) and reverse transcribed using the iScript cDNA synthesis kit (Biorad). For real-time PCR analysis, we used the LightCycler FastStart DNA MasterPlus SYBR Green I kit (Roche) and a Roche Light Cycler 3.5 instrument. Cycle thresholds for each gene were normalized to actin and the results expressed as the fold induction with respect to untreated *HSF1* cells. Statistical significance was determined by an unpaired, two-tailed Student's *t*-test assuming equal variance. Primer sequences are listed in supplementary information, **Table S3**.

### β-galactosidase assays and Western blotting

To monitor Hsf1 transcriptional activity, we transformed yeast cells with a plasmid that expresses the HSE4Ptt-*CYC1-LacZ* reporter. The latter consists of consensus heat shock elements (nTTCnnGAAn)_2_ arranged in a tail-to-tail fashion and inserted upstream of an attenuated *CYC1* promoter that is fused to a *LacZ* reporter gene [53], [89]. β-galactosidase assays were performed using the yeast β-galactosidase assay kit (Pierce, Cat. No. 75768) and relative miller units of expression are shown graphed. For western blotting against Tor1/2, from log phase cultures grown at 25°C, 5 OD_600_ units of cells were collected and proteins extracted by boiling in SDS sample buffer followed by vortexing in presence of glass beads [84], [85]. Extracted proteins were precipitated by TCA, dissolved in 50 mM Tris, pH7.5, 1% SDS and quantified by BCA assay for protein concentration. Equal amounts of protein were denatured using SDS-sample buffer and loaded on a 7.5% SDS-PAGE gel. For obtaining extracts for monitoring Gln3-*myc*_13,_ log-phase cells were treated with rapamycin or methanol and flash frozen. Cell pellets were lysed with glass beads and equal volume of 20% TCA directly as described previously [90], and equal amounts of protein loaded on a 6% SDS-PAGE gel. 12CA5 antibody was used for Gln3-*myc*_13 _detection; anti-Tor1 and Tor2 antibodies obtained from Santa Cruz Biotechnology Inc. were used for detecting Tor1/2 using their recommended procedures.

## Supporting Information

Table S1 (0.09 MB XLS)

Table S2 (0.04 MB XLS)

Table S3 (0.02 MB XLS)

Figure S1 (0.28 MB TIF)
